# How to overcome political and legal barriers to the implementation of a drug consumption room: an application of the policy agenda framework to the Belgian situation

**DOI:** 10.1186/s13722-019-0169-x

**Published:** 2019-11-01

**Authors:** Pierre Smith, Louis Favril, Dominique Delhauteur, Freya Vander Laenen, Pablo Nicaise

**Affiliations:** 1 Institute of Health and Society (IRSS), Université Catholique de Louvain, Clos Chapelle-aux-Champs, 30, 1200 Brussels, Belgium; 2 Institute for International Research on Criminal Policy (IRCP), Faculty of Law and Criminology, Ghent University, Ghent, Belgium; 3 Fondation privée TADAM, Liège, Belgium

**Keywords:** Drug consumption room, Implementation, Drug policy

## Abstract

**Background:**

For more than 30 years, drug consumption rooms (DCRs) have been implemented in Western countries. DCRs are supported by a large body of evidence about public safety and public health effectiveness. However, a political consensus has never been achieved in Belgium on amending the existing law that explicitly penalises the supply of a room for facilitating drug use. Despite this adverse legal and policy framework, a DCR opened in the city of Liège in 2018. In this case report, we applied the theoretical framework proposed by Shiffman and Smith for policy agenda setting, in order to describe and assess how political and legal barriers were overcome in the process of opening the DCR.

**Case presentation:**

For some years, fieldworkers and some city policymakers argued for DCR implementation in Belgium, but without gaining the support of the national authorities, mainly for ideological reasons. In order to address this debate, a feasibility study of DCR implementation in Belgian cities was commissioned. At the national level, an institutional debate took place about the political responsibility for DCRs as a public health intervention, as health care is mainly a matter of regional policy. The lack of consensus led to a situation of political deadlock. Meanwhile, the publication of the study report and the context of local elections offered an opportunity for Liège authorities to reignite the local debate on DCRs. At the local level, law enforcement, care professionals, residents, users, and the press were all involved in the implementation process. Therefore, a local consensus was formed and despite the absence of any national legal change, the DCR opened 1 month before the local elections. It has been working without major medical or legal incident since then. Incidentally, the mayor of Liège was re-elected.

**Conclusions:**

Although the lack of a legal framework may engender instability and affect longer-term effectiveness, the DCR implementation in Liège was successful and was based on a local consensus and effective communication rather than on an appropriate legal framework. The experience provides lessons for other cities that are considering opening a DCR despite an adverse legal and political context.

## Background

Drug consumption rooms (DCRs) are legally sanctioned public health facilities that offer a hygienic environment where people can use pre-obtained drugs in a non-judgemental environment and under the supervision of trained staff [1–3]. They constitute a highly specialised service within a range of services for people who use drugs (PWUD). DCRs are embedded in comprehensive local strategies in order to reach different individuals and fulfil the community needs that arise from illicit drug use [4–7]. DCRs have been operating in Europe, Canada, and Australia for the last three decades. Since the first officially sanctioned facility opened in Berne in 1986, the number of DCRs has risen and has reached more than 90 today [3, 8, 9].

Although DCRs vary in operational procedures and design, their aims are oriented towards public health and safety objectives. In terms of public health, the overall rationale behind DCRs is one of reaching out to, and addressing the problems of, specific high-risk populations of PWUD, especially people who inject drugs and those who consume in public [3]. For this group, DCRs aim to reduce the risk of transmission of blood-borne infections, to reduce the likelihood of morbidity and mortality resulting from overdose, and to help PWUD avoid other harms associated with drug consumption under unhygienic or unsafe conditions [4, 5, 7, 10]. DCRs also aim to reach and maintain contact with socially marginalised groups and to facilitate access to health and social services, including addiction treatment programmes [11–16]. In terms of public safety, DCRs aim to contribute to a reduction in drug use in public places and in the presence of discarded needles and other related public order problems linked with open drug scenes [7, 17].

A substantial body of evidence has accumulated over the past three decades to support the effectiveness of DCRs in achieving their primary health and public order objectives, and therefore supports their role within a continuum of services for PWUD [6, 7, 18]. With regards to legislation, the European Union described DCRs as an effective measure for risk and harm reduction in its *2017*–*2020 Drug Action Plan* [19], as did the International Narcotics Control Board (INCB) in 2016 [20]. An abundance of studies has also demonstrated that feared negative consequences of opening a DCR are not borne out by experience: DCRs do not increase drug use in their vicinity, nor do they encourage young people to initiate drug use [21, 22]. Despite the scientific evidence and international legislation supporting DCRs, there continue to be social and structural barriers to the implementation of this public health intervention in communities across the globe [23, 24]. Accordingly, the debate about implementing new DCRs remains high on the political agenda in a number of countries worldwide, including the United Kingdom and the United States [25–30].

From a legal point of view, international conventions allow flexibility in the establishment of DCRs when national legislation specifically acknowledges that these facilities are part of a public heath, harm-reduction strategy [31]. In several countries, this international framework has been sufficient to permit the implementation of DCRs [32], as was the case in Switzerland in 1986 [33] and in Germany in the mid-1990s [30, 34]. However, other countries were of the opinion that the establishment of DCRs required change in their legislation. That was the case, for example, in France and Luxembourg. In particular, the French legislation was modified in order to allow DCRs in the framework of a medical experiment [35], while new legislation in Luxembourg explicitly excluded DCRs from the articles that punished drug possession and drug-use facilitation [31]. Such exceptions were also introduced in the Canadian legislation [36].

In Belgium, the implementation of DCRs conflicts with the Belgian federal Drug Law of 1921, which makes it a punishable offence to make a room available to facilitate the use of illegal drugs. Since the beginning of the 2000s, several associations of field professionals, local policymakers, and scientific organisations have pleaded for a modification of the law and supported the implementation of DCRs in Belgian cities [37–40]. However, a political consensus has never been achieved on amending the law and allowing implementation. In addition, the Belgian policy system is highly fragmented and complex. While the penal and criminal law is under the responsibility of the federal, i.e. national authority, most prevention and health policy responsibilities have been devolved to the federated authorities (regions and communities), each of which has its own government and majority. It is unclear whether the establishment of DCRs comes under the authority of the federal or federated governments. Against all odds, however, a DCR opened in the city of Liège in September 2018, 1 month before local elections. Against that background, this study aims to assess the drivers that put DCR implementation on the political agenda until its effective implementation, and to look at how the adverse legal and political context was overcome.

We applied the policy agenda framework proposed by Shiffman and Smith [41] in order to organise the presentation of the key elements that favoured the implementation of a DCR in Liège. That framework was initially developed to analyse why some health initiatives receive priority from political leaders and others do not, i.e. the so-called policy agenda setting [42–44]. Shiffman and Smith developed the framework for examining health policy initiatives on maternal mortality in developing countries. Since then, it has been applied to many other health policy fields, including chronic and non-communicable diseases, mental health, and drug policies [45–48]. The framework proposes four categories of key factors: [1] the intrinsic characteristics of the issue, [2] the political context, [3] the power of the actors involved in the policy initiative, and [4] the power of the ideas used to describe the issue (i.e. policy formulation). According to Shiffman and Smith, initiatives are more likely to attract political support when they share specific features in all categories [41]. Firstly, regarding issue characteristics, a health policy initiative is more likely to attract political support when the issue is considered to be sufficiently severe to deserve attention. That severity, however, has to be balanced against other possible priorities that are deemed to be less severe. The importance of an issue is reinforced if credible indicators exist that show clearly both the issue’s severity and interventions that are deemed to be feasible and effective in tackling it. Secondly, the impact of the political context on agenda setting is mainly driven by policy windows, i.e. external conditions that are perceived as favourable for tackling the issue. A textbook example of a favourable policy window is the holding of elections, as they create a context for proposing and discussing new policy initiatives. Thirdly, the policy agenda setting is determined by the power of actors, i.e. the policy cohesion that derives from the existence of strong leadership, guiding institutions that could operate the initiative, and civil society mobilisation. Finally, “the power of ideas” considers the different ways in which actors involved with the issue understand and portray it [49]. Therefore, the power of ideas is based on the capacity of actors to formulate the issue and potential measures to tackle it in a way that makes it possible to achieve a consensus, both internal and external. Internal consensus refers to the level of agreement across stakeholders and the policy community on the definition and terms of the issue. External consensus is the extent to which a consensus is also achieved outside the stakeholder and policy community, e.g. in public opinion and the media. In this study, we have examined these characteristics in the context of the moves to open a DCR in Liège.

This case report results from our involvement in the national feasibility study on DCR implementation in Belgium that was carried out a few months before the opening of the DCR in Liège. During that study, we carried out a series of qualitative interviews with relevant stakeholders (i.e. prosecutorial authorities, law enforcement, local policymakers, and health and social care professionals) as well as PWUD, in the five major cities of the country, including Liège. In addition, we followed up the events that occurred after the publication of the study report and we collected press releases regarding the topic of the potential DCR and subsequently the actual opening of a DCR. Finally, we had additional contacts with the Liège authorities and DCR providers, in order to appraise the context of the opening of the DCR.

## Case presentation

### Local characteristics of the issue

Until the 1960s, Liège, one of the largest industrial cities in Belgium, had thriving mining and steel industries [50, 51]. As happened in many other coal-based industrial areas, its industry declined from the end of the 1970s and the city experienced a socio-economic crisis, with increased poverty and unemployment in some neighbourhoods [52]. The socio-economic decline was accompanied by an increase in illicit drug consumption and trafficking [53]. In addition, it is usually considered that the geographical position of the city, near the Dutch city of Maastricht and the German city of Aachen, is strategic in terms of the illicit drug market [54]. In 2016, in the Walloon region (3.5 million inhabitants) in southern Belgium, the number of heroin users was estimated at 12,000, including 2500 people who inject drugs [55]. Although it is difficult to estimate the size of this hidden and marginalised population, several sources suggest that the size of the DCR target group in the inner city of Liège (200,000 inhabitants) is about 300 PWUD [56]. The geographic landscape of the local drug scene is not spread across the city but is concentrated in the downtown area near the train station, bus station, and shopping malls [31]. In 2013, between 352 and 470 people who inject drugs attended the syringe exchange counters [56]. In 2017, 134,500 syringes were distributed to 550 unique PWUD [56]. The Institute of Forensic Medicine of Liège recorded 35 cases of fatal intoxication by drug use between 2011 and 2013 [56]. The local police drew up 1745 reports related to narcotics in 2016, and 2197 in 2017 [56]. All these figures indicate that the public health and public safety issues related to drug use are becoming more severe.

Over recent years, the City of Liège has developed innovative interventions to tackle issues related to drug use. In 2011, the city supported a 2-year pilot intervention based on heroin-assisted treatment delivery in the form of a clinical randomised controlled trial, called the treatment assisted by diacetylmorphine (TADAM) project [39]. With the approval of the prosecutorial authorities and the “Federal Agency for Medicines and Health Products” (National Belgian medicine agency), in the context of a scientific medical experiment in line with UN drug conventions, a derogation from the federal Drug Law was granted, allowing the medical prescription of diacetylmorphine (heroin) and the availability of a room for its supervised consumption [57]. The pilot project was completed in 2013 and its evaluation showed positive outcomes for PWUD [39, 58]. The only caveat was the cost–benefit analysis, which showed that, compared to methadone maintenance treatment, diacetylmorphine treatment resulted in an annual extra cost per patient of almost €20,000, which was not offset by societal benefits [59]. The limitations of this analysis were, however, that it only involved a small sample of 74 PWUD randomised between the two groups, and its short follow-up period of 12 months. Authorities used this negative cost–benefit analysis and the absence of an amendment to the federal Drug Law to close down the pilot project and the facility [57, 59].

In the light of that experiment, the mayor of Liège undertook several initiatives to support the establishment of a DCR in the city. In particular, in 2014, he introduced a bill in the federal parliament to amend the federal Drug Law in order to permit the establishment of a DCR [60]. However, the bill never reached the level of consensus required. By the end of 2016, the drug policy department of the Belgian Ministry of Public Health published a working paper on DCRs in Belgium [61]. One of the seven conclusions of that paper was that *“if one wishes to implement a DCR, a prior feasibility study is essential. In addition to the above*-*mentioned elements, the budget and legal aspects, including the issues regarding liability of the health care providers and the authorities in case of overdose, must be thoroughly examined*” [61]. In 2017, the feasibility study was carried out in the five largest Belgian cities, including Liège [31]. The aims of this study were [1] to provide an up-to-date overview of the effectiveness of, models of, and barriers to DCRs worldwide, [2] to conduct an analysis of the legal framework for the implementation of a DCR in Belgium, [3] to conduct a qualitative feasibility study with stakeholders (i.e. policymakers, policy administration, law enforcement, criminal justice, drug treatment services, outreach services, and harm reduction services and social welfare services) and PWUD from each of the five Belgian cities, and [4] to formulate recommendations for the implementation of DCRs should they be deemed necessary. The full report of the feasibility study has been published elsewhere [31]. The Liège local authorities used this feasibility study in the development of their policy.

### Political context

The feasibility study showed that most stakeholders in Liège (prosecutorial authorities, law and enforcement professionals, local policymakers, and health and social care professionals), as well as PWUD, agreed that implementing a DCR would be useful [31]. The earlier TADAM experiment was perceived as positive in terms of implementation in the neighbourhood, impact on public safety issues, and PWUD health and wellbeing by most stakeholders. The TADAM experiment also indicated the commitment of local authorities in relation to drug use issues in the city since the early 2000s (i.e. local policymakers and law enforcement). Indeed, when considering several technical options for the establishment of a DCR in their city, e.g. regarding opening hours, inclusion/exclusion criteria, and the type of personnel needed, all stakeholders interviewed in the feasibility study referred positively to the previous TADAM experience [31]. The publication of the national feasibility study in February 2018 [31] was used by the mayor of the city of Liège as an opportune political window to reignite the political debate on DCRs in the context of local elections. In the same period, press articles were published about the drug scene in the city of Liège (on the numbers of drug users and the prevalence of drug-related harm, consumption in public spaces, discarded syringes, and drug-related crime), as were articles commenting on the results of the feasibility study [54, 62]. Against that background, the mayor organised several meetings in the city to discuss both his political programme for the local elections and the results of the feasibility study [63, 64]. Several meetings were organised with the local prosecutorial authorities and the police in order to anticipate legal and security issues. Meetings also took place with residents, in order to discuss the potential effects of a DCR on the health and welfare of PWUD, as well as on public safety and nuisances related to drug use in the city centre, bearing in mind the earlier positive TADAM experience. These meetings were organised with the participation of different local policymakers, including from the opposition and, despite the electoral context, which might have favoured confrontation, a broad political consensus was reached across all the parties represented on the city council in support of the DCR [64].

### The power of actors

Fragmentation of policy responsibilities is one of the key features of the Belgian policy system [65, 66]. In particular, health policy responsibilities are shared within a complex system of different policy authorities: the federal (national) authority and several levels of federated authorities (regions and communities, whose territories overlap in some cases). A single health policy decision usually requires the agreement of at least two different authorities. By contrast, public safety is mainly the responsibility of the federal authorities, although local authorities have extensive autonomy for local initiatives. As a result, decision-making usually involves lengthy negotiation processes between diverse stakeholder groups and authorities, and decisions are driven by a high level of corporatism, with each segment aiming to protect its particular interests [66]. In addition, the Belgian policy system is characterised by a lack of cohesion, by multiple, sometimes competing, guiding institutions, and by weak overall leadership [67]. At the same time, this kind of system also leaves extensive room and autonomy for local initiatives and local leadership.

During the 2014–2019 legislature, no consensus was reached on new drug policy initiatives at the federal level [68]. This meant that there was no support for DCRs or for an amendment to the Drug Law from the federal government during the legislature. The Belgian federal government is usually formed by coalitions made up of different parties. Although some of the parties were not opposed to DCRs and were keen to consider the scientific evidence on their effectiveness, some other parties made their reluctance clear. Traditionally, when there is no consensus among members of the government on social issues, no decision is made [66], leading to a political deadlock. The situation, however, left room for local authority initiatives. Several local policy stakeholders pleaded for change, in particular regarding the possibility of implementing DCRs and other harm-reduction initiatives, particularly in the French-speaking community [69–71]. In the city of Liège, the local authorities had, since 2003, been developing a local drug policy plan that emphasised the need for a DCR in the city [72, 73]. In addition, the mayor had publicly taken the lead on a policy that favoured the opening of a DCR.

### The power of ideas

The key policy formulation regarding DCRs is related to their ambiguous position between the penal and public health domains. Within the federal structure of the Belgian state, the Belgian Drug Law (1921), which is a penal law, is under the responsibility of the federal, i.e. national authorities, while the main responsibility for health policy, including prevention and harm reduction, is regional. After taking note of the lack of political willingness to amend the penal law at the federal level, the mayor of Liège obtained the support of the Walloon regional authority in the form of a resolution acknowledging that DCRs are an effective tool for harm reduction [74, 75]. As a result, an institutional debate took place about the potential conflict of policy responsibilities between the regions and the federal authorities. Some policymakers in the Brussels region also proposed regional regulation of harm-reduction interventions, including DCRs, arguing that health was a regional competence [76]. A formulation of policy on the issue that clearly presented DCRs as an instrument for public health led to an internal consensus among French-speaking stakeholders (in Brussels and Wallonia). This policy formulation helped to block possible counter-initiatives against the implementation of the DCR, e.g. from the federal authorities. Interestingly, however, the external consensus, i.e. involving the local stakeholders and residents, was achieved by emphasising the benefits of a DCR in terms of tackling public safety nuisances. Local authorities were successful in presenting the policy issue and intervention using both the public health and public safety frameworks.

### The establishment of the DCR

Taking into account all the elements analysed within the four categories of Shiffman and Smith’s framework, the outcome was the opening of a DCR in Liège in September 2018, 1 month before the local elections, despite the adverse legal and federal policy framework. The DCR is funded by the city authorities and the Walloon region and is coordinated by the TADAM foundation that had managed the previous diacetylmorphine-assisted treatment project. The DCR was established in the same premises, which already had the appropriate infrastructure, including eight injection booths and one room for drug smoking for twelve PWUD. These premises are located in the downtown area, close to the local drug scene. Before the opening of the DCR, a consultation with local stakeholders was organised in order to validate the different operating criteria for the DCR (PWUD admission criteria, intake procedure, registration procedure, opening hours, house rules and regulations, and staff required), based on the previous TADAM experience and the results of the feasibility study [31, 56]. The public prosecutor’s office published a note explaining that it would not initiate prosecution against the DCR, its staff, or its users, although it would decide, depending on the circumstances, whether to prosecute the institution in the event of a complaint from a third party (public or civil party) [77]. During the week before the opening, the residents of the area were invited to visit the room and the press reported positively on the event [63]. Incidentally, the mayor was re-elected in October 2018.

As previously explained, the DCR’s target public was estimated at 300 PWUD [56]. Preliminary data gathered by the DCR providers stated that after 3 months, the DCR had reached 72% (216 PWUD) of its estimated target public, and 121% (363 PWUD) after 6 months, without any major medical or legal incident. Over the first 6 months, the average number of daily visitors was 35 (range 4–78). Of all registered visits (N = 6292), drugs were injected on 42% of visits and inhaled on 55%. The main drugs consumed were heroin (74% of visits) and cocaine (20% of visits). In addition, 44 PWUD have changed their route of administration from injection to inhalation. Two overdose events were adequately managed; these required resuscitation by the DCR staff and evacuation to an emergency department, without severe consequences.

## Discussion and conclusions

Despite the lack of political consensus at the national level and the lack of a legal framework, a lack that might lead to instability and affect longer-term effectiveness, the DCR implementation in Liège has been successful. The four categories of Shiffman and Smith’s framework help to clarify how the local characteristics of the issue, the policy context, the power of actors, and formulation of policy on the issue contributed to this outcome. In the specific context of a long history of initiatives in relation to harm reduction in Liège, the implementation of the DCR was made possible, in the context of the local elections, by a consensus among local stakeholders (policymakers from the different parties, the public prosecutor’s office, law and enforcement professionals, health and social care professionals, and residents) and by a communication strategy that involved all these stakeholders and researchers. The local initiatives were feasible thanks to the commitment of the local authorities and the context of fragmentation and political deadlock at the national level.

The literature makes it clear that the lack of political consensus in national governments favouring the implementation of DCRs is mainly related to issues of morality, ideology, public acceptance, and negative media response and the associated political consequences [30, 78–81]. The strategy in Liège, however, proved to be successful in overcoming barriers of that kind. What is more, the local political support for DCR implementation was, in this case, a factor of popularity in the context of the elections.

One key element for the opening of the DCR has probably been the local consensus of stakeholders (prosecutorial authorities, law enforcement professionals, local policymakers, health and social care professionals, and residents). This consensus was made possible by the visible increase in public safety issues related to drug use over the last decade in the city and by the past experience of harm-reduction interventions, i.e. the TADAM pilot project. Local stakeholders and the population all agreed that the TADAM experience had been beneficial and criticised its closure [31]. A second key element was the communication strategy and the consultation of local stakeholders, leading to their involvement throughout the process of implementing the DCR. The publication of the feasibility study, which recalled the evidence on DCRs, and the context of the local elections helped to create momentum. The Vancouver experience also made it clear that one element of success in the implementation of their DCR was the consultation and involvement of key stakeholders, including the residents, in the process of establishing the DCR [82]. Indeed, consulting local stakeholders makes it possible to establish collaboration and a dialogue on the resolution of issues and misunderstandings in relation to DCRs [82]. The emphasis on public nuisances (consumption in public spaces, discarded syringes, and drug-related crime) in the formulation of the policy issue may have contributed to the involvement of local stakeholders and residents. In the Netherlands, the majority of DCR managers explained that the primary rationale for founding a DCR was the reduction of public nuisances [83, 84]. In contrast, a policy formulation clearly presenting DCRs as a public health instrument has led to an internal consensus between policymakers at the level of the federated authorities.

Finally, the lack of political consensus at the national level left room for local leadership and autonomy for initiatives, in particular where there was a prior history of similar interventions. This kind of context has also been observed to an extent in France, for example. The case made by the Paris City Council for the opening of a DCR in 2010 and 2013 led to a compromise at the national level, with the French parliament adopting a new law that permitted DCRs as an experiment in 2016 [35, 85]. In Vancouver in 2003, the exemption granted by the then Canadian government, which allowed the opening of the DCR, followed the election of a new mayor in 2002, Larry Campbell, who had made the opening of a DCR in Vancouver a key part of his programme [36, 86]. However, this bottom-up development of initiatives requires local powers and autonomy, which do not exist in all countries. For example, one study observed that the absence of DCRs in the UK, even though some cities wanted to open them, may reflect the limited power and autonomy of local authorities as compared to other countries, such as Germany [30].

However, the Liège initiative remains unstable and the lack of an appropriate legal framework may weigh on its long-term effectiveness. It remains unclear whether the courts would interpret the article of the federal Drug Law that punishes the facilitation of a room for drug use in a way that would rule out DCRs. The outcome of any such case remains unknown. The development of Belgian drug policy is characterised by a bottom-up approach, in which innovative interventions have often been initiated at a local level [87] and have only later become part of a legal framework. This has been particularly the case for harm-reduction interventions such as opioid substitute treatments [88], syringe exchange [89], and the organisation of drug-addiction treatments in prisons [90]. These harm-reduction interventions had no legal framework or were even illegal when they were implemented, but they were legalised or regularised over time. This might also happen to DCRs in Belgium. Since the DCR was established in Liège, other Belgian cities, particularly in the French-speaking community, have developed plans for establishing their own DCRs in the near future, including Brussels, Charleroi, and Namur [91–93]. In Charleroi, the city council came out in favour of the implementation of a DCR [93]. Finally, Brussels, which has its own regional government, approved a draft decree providing for the implementation of a DCR in the city during the next legislature as part of a broader, integrated regional drug policy [91, 94].

## Data Availability

Not applicable.

## Abbreviations

DCR: drug consumption room
HIV: human immunodeficiency virus
HVC: hepatitis C virus
PWUD: people who use drugs
TADAM: treatment assisted by diacetylmorphine
